# Amplicon Sequencing Reveals Rhizosphere Fungal Dysbiosis Facilitates Goji Berry Root Rot Onset

**DOI:** 10.3390/plants14213325

**Published:** 2025-10-30

**Authors:** Tianyu Wang, Yao Chen, Meng Yan, Haonan Wang, Kai Guo, Xudong Zhou, Hexing Qi, Lifeng Zhou

**Affiliations:** 1 State Key Laboratory for Development and Utilization of Forest Food Resources, Zhejiang A&F University, Hangzhou 311300, China; 2023602121107@stu.zafu.edu.cn (T.W.); 2023602122008@stu.zafu.edu.cn (Y.C.); 2024602122123@stu.zafu.edu.cn (M.Y.); haonanwang@zafu.edu.cn (H.W.); kaiguo@zafu.edu.cn (K.G.); xudong.zhou@zafu.edu.cn (X.Z.); 2 College of Agriculture and Animal Husbandry, Qinghai University, Xining 810016, China; qhx390495559@126.com

**Keywords:** microflora, species abundance, ecological diversity, arid climate

## Abstract

Root rot in *Lycium barbarum*, an economically vital crop, is a critical barrier to its sustainable development in China. To elucidate the underlying micro-ecological mechanisms, this study aimed to characterize and compare the rhizosphere microbial communities of healthy and diseased plants from the Qaidam Basin. We employed PacBio full-length amplicon sequencing to analyze bacterial and fungal populations, complemented by network analysis and in vitro antagonistic assays. The results indicated that while microbial species richness was similar, the community structures of healthy and diseased soils were fundamentally different, suggesting that the disease is primarily driven by microbial dysbiosis rather than species loss. Healthy soil was enriched with beneficial *Trichoderma*, whereas diseased soil was dominated by the pathogen *Fusarium*, with an abundance 6.7 times higher than that in healthy soil. Network analysis revealed the healthy fungal community was significantly more stable (modularity index: 0.818) than the diseased network (0.4131), where *Fusarium* occupied a core hub position. Crucially, *Trichoderma* strains isolated from healthy soil exhibited strong antagonistic activity against *Fusarium*, with an average inhibition rate exceeding 75%. This study identifies *Fusarium* as the key pathogen of Goji root rot and native *Trichoderma* as a potent biocontrol agent, providing a scientific basis for a sustainable, micro-ecological control strategy.

## 1. Introduction

*Lycium barbarum*, also known as Goji berry, is a perennial deciduous shrub in the Solanaceae family [1] that produces fruits a prized as a traditional Chinese medicinal material [2]. The berry possesses a wealth of activities [3], including anti-aging [4], neuroprotective and anti-fatigue effects [5], as well as promoting metabolism, regulating blood sugar [6], alleviating glaucoma symptoms, providing antioxidant benefits [7], modulating the immune system [8], and exhibiting anti-tumor properties [8]. Furthermore, its high tolerance to drought [9], salinity [10], and temperature extremes [11,12] makes it a vital plant for ecological restoration and desertification control in arid regions [13].

There are approximately 80 wild Goji species worldwide, primarily distributed in the temperate to subtropical regions of South America and Eurasia. In China, seven species and two varieties have been identified [14], mainly in northern regions such as Ningxia, Qinghai, Gansu, Hebei, Inner Mongolia, and Xinjiang. Among these, Qinghai Province [15], located in the northeastern part of the Tibetan Plateau, has the largest Goji cultivation area in China, reaching 49,300 hectares [16]. Due to this combination of values, the Goji berry industry has expanded significantly [15,16], with China’s annual production exceeding 420,000 tons and exports reaching over 40 countries [13]. However, with the promotion of intensive cultivation models, especially with increasing years of continuous cropping, soil-borne diseases, primarily root rot, have become increasingly rampant, severely threatening the yield and quality of Goji berries. This disease, colloquially known as ‘goji berry cancer’, causes devastating losses, with incidence rates reaching over 50% in some plots in the Qaidam Basin [17]. While its pathogenic mechanism remains unclear, previous studies consistently point towards multiple species of the fungus *Fusarium* as the primary causal agents [18]. Previous studies have indicated that the primary pathogens of Goji root rot are multiple species of *Fusarium*, including *Fusarium acuminatum*, *F. oxysporum*, *F*. *solani*, and *F. equiseti*, with *F. oxysporum* and *F. solani* being frequently reported as the main pathogens [18]. The genus *Fusarium* is widespread in agricultural soils and possesses strong environmental adaptability. Its dormant chlamydospores can survive in the soil for long periods and rapidly activate to cause disease upon encountering a suitable host and environmental conditions. As a high-value crop, Goji berry is commonly cultivated under intensive continuous cropping models in major production areas like the Qaidam Basin. The intensive continuous cropping model is an agricultural practice characterized by growing the same crop or multiple crops on the same piece of land repeatedly, year after year, with minimal or no fallow periods, combined with intensive farming methods. However, as has been widely reported in various crop systems, long-term continuous cropping leads to ‘continuous cropping obstacles’, a core feature of which is the progressive degradation of the soil micro-ecological environment. The current reliance on chemical fungicides offers limited, short-term control and raises concerns about pathogen resistance and ecological disruption. Therefore, there is an urgent need to explore sustainable, microbe-centered control strategies built on a deeper understanding of the root-zone ecology [19].

In recent years, researchers have increasingly recognized that regulating the rhizosphere microbial community structure, increasing the proportion of beneficial microorganisms, and constructing an antagonistic micro-ecosystem are crucial directions for the green and sustainable control of soil-borne diseases [20]. This finding has provided a new theoretical framework for the study of plant–soil-borne diseases and has spurred the development of microbiome-based disease control strategies [21]. It has been successfully applied to elucidate the root rot mechanisms in several cash crops, such as *Panax notoginseng* [22], *Panax quinquefolius* [23], and *Nicotiana tabacum* [24]. Soil microorganisms are core factors in maintaining the functionality of soil ecosystems, and the diversity and composition of their communities directly influence ecosystem multifunctionality [25]. The concept of “disease-suppressive soil” has been proposed, referring to soils that naturally possess the ability to inhibit specific diseases, a mechanism closely linked to specific beneficial microbial communities. As the stability and complexity of networks are key to maintaining soil health [26], contemporary microbial ecology research has moved beyond simple species composition analysis to focus on the interaction networks between species.

Modern microbiome research is greatly enhanced by high-throughput sequencing technologies [27]. In this study, we employed PacBio long-read sequencing, which provides full-length coverage of phylogenetic markers like the bacterial 16S rRNA gene and the fungal ITS region [28]. This approach offers the potential for higher taxonomic resolution compared to short-read technologies, which typically only cover variable sub-regions of these markers [29]. Obtaining full-length amplicon data provides a more accurate foundation for species-level classification, which is crucial for distinguishing between pathogenic and beneficial microbes and exploring the ecological mechanisms of disease suppression [30]. Third-generation high-throughput sequencing technology has provided powerful support for microbiome research [27]. Through parallel sequencing and deep coverage, it can efficiently resolve the structure and diversity of microbial communities in complex samples [28]. The generation of amplicon sequencing data lays a critical foundation for revealing the biological functions and ecological roles of microorganisms, helping to deeply analyze the compositional characteristics and dynamic changes in microbial communities and providing data support for exploring microbial ecological regulation mechanisms [29]. By combining high-throughput sequencing with the screening of biocontrol agents, it is possible to systematically reveal the ecological niche characteristics and community dynamics of pathogens, providing a theoretical and practical basis for new micro-ecological control technologies [30]. The primary goal of this study was to elucidate the key microbial drivers of Goji berry root rot disease in the Qaidam Basin. To achieve this, we defined the following objectives: (1) use PacBio full-length amplicon sequencing to compare the bacterial and fungal community differences between healthy and diseased Goji rhizosphere soils; (2) investigate the relationship between microbial community structure changes and the occurrence of root rot, identifying key pathogenic and disease-suppressive microbial taxa; (3) isolate and verify local microbial resources with antagonistic activity and provide candidate strains for the development of targeted biological agents.

## 2. Results

### 2.1. Sequencing Data Overview

PacBio amplicon sequencing was performed on 12 rhizosphere soil samples, comprising six from healthy plants (H1–H6) and six from diseased plants (D1–D6). After stringent quality control and processing, a total of 369,549 high-quality fungal ITS sequences (average length 626 bp) and 338,836 high-quality full-length bacterial 16S rRNA sequences (average length 1490 bp) were obtained. These sequences were clustered into 820 fungal ASVs and 2695 bacterial ASVs. The detailed sequencing statistics for each sample, including raw and clean read counts, base numbers, average lengths, and ASV counts, are presented in Table 1.

### 2.2. Root Rot Structurally Alters Microbial Community Diversity

**Figure 1:**
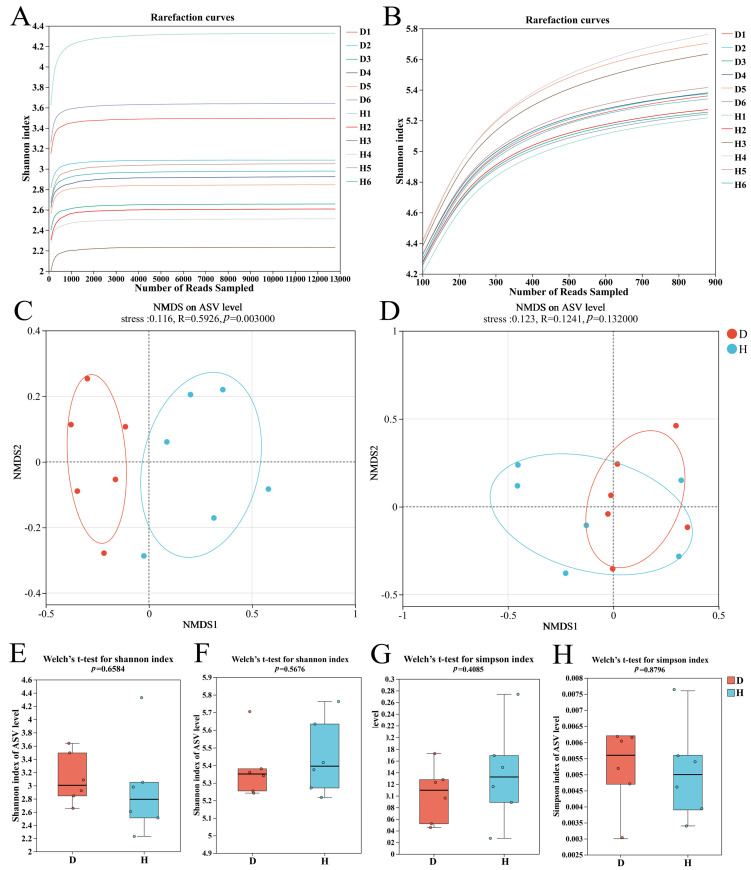
Diversity analysis of Goji rhizosphere soil. (**A**) Rarefaction curves for fungal samples (D1–D6: diseased group; H1–H6: healthy group); (**B**) rarefaction curves for bacterial samples; (**C**) NMDS analysis of fungal communities (the stress value of <0.2 indicates a reliable ordination); (**D**) NMDS analysis of bacterial communities (D, diseased groups; H, healthy groups); (**E**,**F**) Shannon index analysis for fungal and bacterial communities, where higher values indicate greater diversity; (**G**,**H**) Simpson index analysis for fungal and bacterial communities, where lower values indicate greater diversity. In the boxplots (**E**–**H**), the center line indicates the median, the box represents the interquartile range (IQR), and the whiskers extend to 1.5 times the IQR. Individual points are outliers.

To assess the impact of root rot on microbial diversity, both alpha and beta diversity analyses were conducted (Table 2). Rarefaction curve analysis for both fungal (Figure 1A) and bacterial (Figure 1B) communities showed that the curves approached a plateau, indicating that the sequencing depth was sufficient to capture the majority of the microbial diversity present in the samples. Alpha diversity analysis, which measures within-sample diversity, revealed a striking result. As shown in Figure 1E,H and Table 1, there were no statistically significant differences (*p* > 0.05) in the Shannon, Simpson, or Chao1 indices between the healthy (H) and diseased (D) groups for either the fungal or bacterial communities.

In sharp contrast to the alpha diversity results, beta diversity analysis, which measures between-sample community differentiation, revealed a profound shift in community structure. The NMDS plot based on Bray–Curtis dissimilarity (Figure 1C) showed a clear and distinct separation between the fungal communities of the healthy and diseased groups. This visual separation was statistically confirmed by ANOSIM, which yielded a highly significant result (R = 0.5926, *p* = 0.003). Conversely, the bacterial communities from the healthy and diseased groups showed considerable overlap in the NMDS plot (Figure 1D), and the ANOSIM test confirmed that there was no significant difference between them (R = 0.1241, *p* = 0.132).

### 2.3. Community Composition and Key Differential Taxa

**Figure 2:**
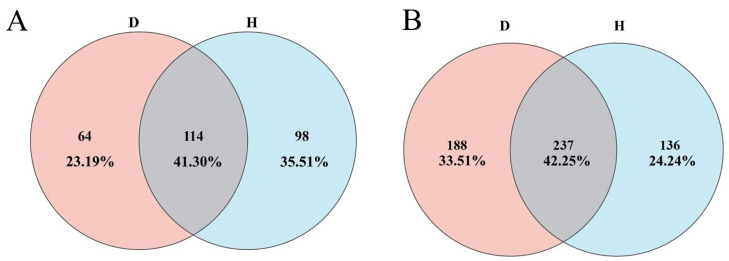
Venn diagrams of shared and unique ASVs. (**A**) Fungal community ASVs; (**B**) bacterial community ASVs. D, diseased groups; H, healthy groups.

To understand the nature of the community restructuring, the study analyzed the taxonomic composition. Venn diagrams (Figure 2) revealed differences in the number of unique species. The healthy fungal community contained more unique ASVs than the diseased one (98 vs. 64), whereas the diseased bacterial community harbored more unique ASVs than the healthy one (188 vs. 136).

**Figure 3:**
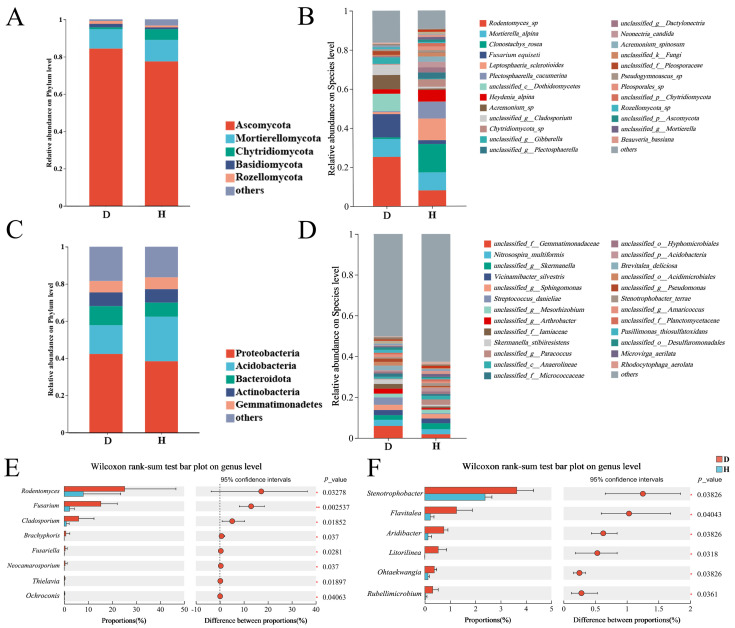
Taxonomic composition of Goji rhizosphere soil communities and differential abundance analysis of microbial genera. (**A**) Fungal community composition at the phylum level; (**B**) bacterial community composition at the phylum level; (**C**) fungal community composition at the species level (top 30); (**D**) bacterial community composition at the species level (top 30); (**E**) significantly different fungal genera between healthy and diseased groups; (**F**) significantly different bacterial genera. D, diseased groups; H, healthy groups. In the bar charts (**A**,**C**), colored segments represent the relative abundance of microbial phyla. In the Wilcoxon plots (**E**,**F**), bars show the difference in mean proportions, error bars represent 95% confidence intervals, and circles indicate individual sample values.

At the phylum level (Figure 3A), the fungal communities in all samples were dominated by Ascomycota (average relative abundance 80.90%), followed by Mortierellomycota (10.99%) and Chytridiomycota (3.34%). The bacterial communities were dominated by Proteobacteria (40.35%), Acidobacteria (19.72%), Bacteroidota (8.95%), Actinobacteria (7.31%), and Gemmatimonadetes (6.25%) (Figure 3C). The most revealing differences emerged at the species and genus levels. Analysis of the top 30 most abundant fungal species (Figure 3B) showed that while some species like *Mortierella alpina* were abundant in both groups, the diseased group was characterized by a high abundance of *Fusarium equiseti* (11.93%). In contrast, the healthy group was dominated by taxa such as *Clonostachys rosea* (14.49%). Wilcoxon rank-sum test (Figure 3E) confirmed these observations at the genus level, identifying *Fusarium* as the most significantly enriched genus in the diseased group. Its relative abundance was 6.7 times higher in diseased soil compared to healthy soil (*p* < 0.01). Conversely, the genus *Trichoderma*, a well-known biocontrol agent, was significantly enriched in and almost exclusively detected in the healthy soil samples. In contrast, no bacterial genera showed such a strong and consistent differentiation between the two groups (Figure 3D,F). These results indicated the proliferation of *Fusarium* and the depletion of *Trichoderma* as the key microbial signatures of Goji root rot.

### 2.4. Co-Occurrence Network Analysis Reveals a Pathogen-Dominated, Destabilized Community

**Figure 4:**
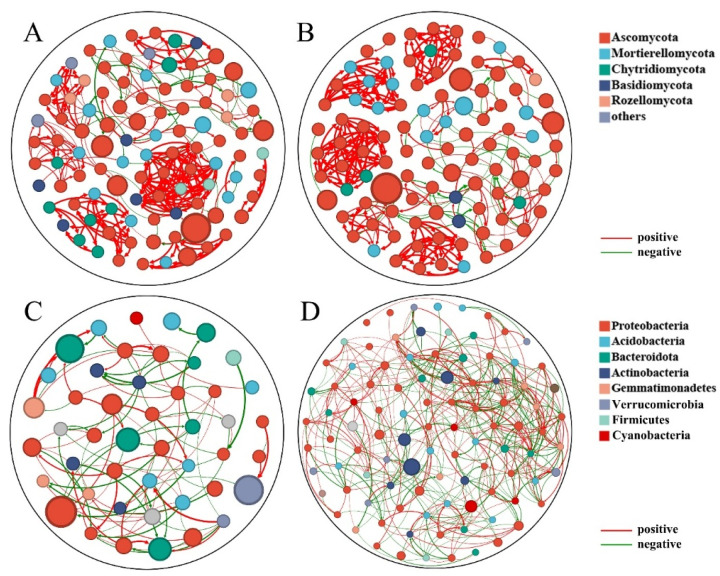
Co-occurrence network analysis of fungal and bacterial communities. (**A**) Diseased fungal network. (**B**) Healthy fungal network. (**C**) Diseased bacterial network. (**D**) Healthy bacterial network. Node size represents relative abundance. Lines represent significant Spearman correlations (positive or negative).

Based on differential species analysis, there was a highly significant difference in the abundance of *Fusarium* between healthy and diseased Goji rhizosphere soils, suggesting it might be the cause of Goji root rot. To investigate the interaction patterns between species, the study constructed co-occurrence networks. The network topological parameters (Table 3) showed that the healthy fungal network had a significantly higher modularity (0.818) compared to the diseased fungal network (modularity = 0.413). In network ecology, high modularity is generally positively correlated with the stability of a network and its resistance to external disturbances, such as pathogen invasion. This indicated that the fungal community structure in healthy soil was more stable and its functional divisions were more distinct. After the onset of disease, this stable modular structure collapsed, and the network became simpler and more fragile (Figure 4A,B).

Visualization of the networks (Figure 4) made these structural differences apparent. The healthy fungal network (Figure 4B) is characterized by four distinct, tightly clustered modules, indicative of a resilient community structure. The diseased fungal network (Figure 4A), on the contrary, has coalesced into a single, large, less-defined module. Critically, the analysis of node-level importance revealed the ecological role of *Fusarium*. The ASV representing *Fusarium* (ASV27) emerged as a key hub node in the diseased network, possessing extremely high centrality (ranked 2nd) and PageRank values (ranked 3rd). In network theory, such nodes are considered “keystone species” that exert a disproportionately large influence on the entire network’s structure and function. This finding provides powerful ecological evidence that *Fusarium* is not merely abundant but acts as a key driver of the community shift, actively restructuring the network into a pathogenic state. In the healthy network, this same node was of negligible importance. In the bacterial network, however, the difference in modularity was not significant, suggesting that the stability of the bacterial network was not associated with the disease state of the Goji plants.

### 2.5. In Vitro Assays Functionally Validate the Antagonistic Relationship

**Figure 5:**
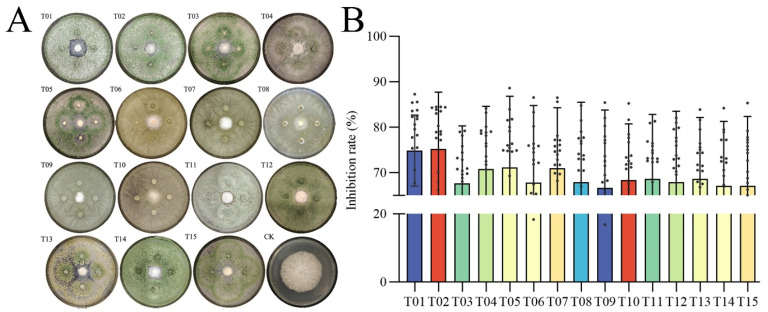
*Fusarium* abundance and antagonism. (**A**) Images of the inhibition of *Fusarium* by *Trichoderma* groups after 72 h of co-culture. (**B**) Inhibition rate of the 15 *Trichoderma* groups against 18 *Fusarium* groups. In plot (**B**), bars represent the mean inhibition rate for each *Trichoderma* group against all tested *Fusarium* groups, error bars indicate the standard deviation, and individual points show the inhibition rate against each of the 18 *Fusarium* isolates.

Based on the colonies, conidiophores and conidia morphological characteristics, 18 *Fusarium* groups and 15 *Trichoderma* groups, were obtained from Goji rhizosphere soil samples. Subsequently, plate confrontation assays between the 18 *Fusarium* groups and the 15 *Trichoderma* groups were conducted (Figure 5A), and the average inhibition rates were calculated (Figure 5B). The results revealed that the 15 *Trichoderma* groups exhibited broad inhibitory effects against all the 18 *Fusarium* groups, with a high average inhibition rate of 75.3%, and a maximum inhibition rate exceeding 88%. This finding indicated the antagonistic relationship between *Trichoderma* and *Fusarium* groups.

## 3. Discussion

The rampant spread of root rot in major Goji berry production regions has become a critical bottleneck restricting the sustainable development of this unique industry. The conclusion of this study is that the outbreak of Goji root rot is not caused by a simple loss of microbial species or the isolated presence of a pathogen, but is strongly associated with a profound rhizosphere microbial community dysbiosis. The hallmark of this imbalanced state is a fundamental reshaping of the microbial community composition (β-diversity) without a significant change in species richness (α-diversity). This ecological shift is primarily driven by changes within the fungal community, which undergoes a dramatic reorganization, while the bacterial community remains relatively stable. Specifically, a simplified, pathogen-dominated, disease-conducive community has replaced the original, stable, healthy community. Through high-throughput sequencing and microbial network analysis, this study precisely identified the two key players in this ecological dysbiosis. In diseased soil, the relative abundance of the pathogenic fungus *Fusarium* became 6.7 times higher than in healthy soil, making it the absolute dominant taxon. In stark contrast, the fungus *Trichoderma*, which is a promising candidate for investigation as a biocontrol agent, was specifically enriched in healthy soil. In vitro antagonism experiments further confirmed that *Trichoderma* strains isolated from healthy soil exhibited an average inhibition rate of 75.3% against *Fusarium* pathogens isolated from diseased soil, suggesting a strong antagonistic relationship between the two.

In the Goji berry root rot ecosystem of the Qaidam Basin, *Fusarium* appears to be not only the most abundant pathogen but may also plays the role of a “key pathogen,” with pathogenic effects manifested on two levels: direct attack on the host plant and indirect disruption of the soil micro-ecosystem. Through isolation, culturing, and identification, this study identified *Fusarium* spp. as the main pathogenic species, which is consistent with findings from other Goji production areas [31]. *Fusarium* species are typical soil-borne pathogens, and their pathogenic process follows the classic vascular wilt model. First, *Fusarium* has extremely strong environmental adaptability and can survive in the soil for several years as dormant structures like chlamydospores, awaiting a suitable host and environmental conditions [32]. Once specific signal substances secreted by Goji roots are detected, the dormant spores germinate, and their hyphae grow chemotactically towards the roots. Second, the hyphae typically invade the root epidermis through natural openings such as root tips and lateral root formation sites, or through microscopic wounds caused by nematodes or agricultural practices [33]. After invasion, the hyphae grow intercellularly for a short period in the root cortex and quickly reach the vascular system [34]. Finally, the most critical pathogenic step is the colonization and spread of *Fusarium* within the xylem vessels. Upon entering the vessels, the fungus produces a large number of microconidia, which are passively transported upwards with the plant’s transpiration stream, achieving systemic infection of the entire plant [33]. The proliferation of hyphae within the vessels, along with defensive substances like gels and tyloses produced by the plant to resist pathogen spread, collectively cause a physical blockage of the xylem vessels [35]. This blockage severely impedes the upward transport of water and mineral salts, ultimately leading to typical symptoms in the plant’s aerial parts, such as wilting, leaf yellowing, stunted growth, and eventually death, which matches the root rot symptoms observed in the field in this study.

The network analysis results of this study showed that the ASV representing *Fusarium* had high centrality and PageRank value in the network of diseased communities. In network theory, this means that the node has a decisive impact on the structure of the entire network [36]. Therefore, the study identified *Fusarium* as the key pathogen, and its ecological impact was far greater than would be suggested by its abundance alone. Healthy microbial community networks usually have the characteristics of high complexity and high modularity, which means that there are many closely interacting microbial communities in the network. This structure enhances the stability of the ecosystem and the resistance to external interference. This study found that the modularity index of healthy soil fungal network was 0.818, while the diseased soil network decreased sharply to 0.4131, indicating that the presence of *Fusarium* had broken the original balance of microbial interaction, leading to the disintegration of network function modules and the simplification of network structure. This simplified network is more fragile, more prone to collapse under environmental stress, and more likely to cause a series of secondary diseases. This finding is consistent with the research results of other soil borne diseases; that is, the occurrence of diseases is often accompanied by the reduction in microbial network complexity and the loss of negative correlation connection with regulatory effect [37]. The ecological damage was further magnified under the agricultural background of continuous cropping of *L. barbarum*. The long-term cultivation of single crops provides a continuous host and nutrient source for *Fusarium*, leading to its selective enrichment in soil, which is called “continuous cropping obstacle” [38]. This study revealed the ecological consequences of this process: continuous cropping first screened and enriched *Fusarium*; when its abundance crosses a critical point, *Fusarium* will actively inhibit and eliminate other competitors and beneficial microorganisms by virtue of its competitive advantage, eventually leading to the collapse of the entire microbial network. This formed a vicious circle: continuous cropping leads to the enrichment of *Fusarium*, which leads to the instability of the ecosystem, and the unstable ecosystem cannot inhibit *Fusarium*, thus making the disease more severe. This also explains why chemical fungicides often have a limited effect and are prone to relapse because they only kill pathogens [39] and fail to repair the damaged soil micro-ecosystem that has lost its self-regulation ability.

In contrast to the destructive role of *Fusarium*, the *Trichoderma* found in healthy rhizosphere soil in this study demonstrates a powerful ability to build and maintain a disease-suppressive soil as a potential “key beneficial fungus.” *Trichoderma* is such an effective biocontrol agent because it has evolved a multi-dimensional, synergistic set of antagonistic mechanisms, covering direct attack, chemical warfare, and resource competition. The inhibition rate of 75.3% observed in this study might be the result of the combined effect of these mechanisms. Mycoparasitism is *Trichoderma*’s most direct and aggressive weapon. *Trichoderma* hyphae can recognize and grow towards the hyphae of pathogens like *Fusarium*, then tightly attach to them by coiling and forming structures like appressoria [39]. Subsequently, *Trichoderma* secretes a suite of cell wall-degrading enzymes, mainly chitinases and β-1,3-glucanases, which efficiently hydrolyze the chitin and glucan that form the backbone of the fungal cell wall [40]. Once the cell wall is dissolved, *Trichoderma* hyphae can invade the pathogen’s body, absorb its nutrients, and ultimately cause the pathogen’s cells to lyse and die. This process provides a direct mechanistic explanation for the clear inhibition zones observed in our plate confrontation assays. The specific presence of *Trichoderma* in healthy soil and its multifaceted antagonistic capabilities observed in vitro make it a strong candidate for the definition of a “key beneficial fungus” or “disease-suppressive factor.”

Synthesizing all the findings of this study, the occurrence of Goji berry root rot appears to be strongly influenced by the interplay between pathogenic and antagonistic fungi in the Goji berry rhizosphere soil. The intensive monoculture model of Goji berry cultivation leads to the continuous accumulation of pathogens in the soil environment, which ultimately emerge as keystone pathogens capable of degrading network stability and steering the entire ecosystem towards a “diseased state.” Conversely, a network characterized by the presence of key beneficial fungi, such as *Trichoderma*, forms a complex, stable microbial web replete with antagonistic relationships that can effectively suppress the proliferation of pathogens. These indigenous strains possess a natural advantage in environmental adaptability. The immediate next step should be the development of these strains into localized bio-fungicides or bio-organic fertilizer products. Application of these products during the initial stages of disease or as a preventive measure is expected to directly suppress the *Fusarium* population through a “microbe-controls-microbe” strategy. To solve the problem, we should focus on the restoration and reconstruction of the whole soil ecosystem. This requires a set of comprehensive management measures, whose core goal is to regulate the composition of soil microbial community. To construct a healthy soil ecosystem capable of self-regulation and repair, a comprehensive strategy is necessary. This approach begins with the regular application of *Trichoderma* biological agents to maintain a dominant population of beneficial microorganisms. Concurrently, increasing the use of high-quality organic fertilizer is essential, as it provides the necessary food and habitat to support a diverse microbial community, thereby improving the ecosystem’s overall complexity and stability. Furthermore, agricultural practices should be adapted to reduce the accumulation of pathogens like *Fusarium* in the soil through the implementation of sensible crop rotation or intercropping systems. Finally, optimizing irrigation management to avoid conditions of long-term soil saturation or hardening creates an environment that is hostile to pathogenic bacteria. The synergistic combination of these measures aims to cultivate a resilient and balanced soil environment.

## 4. Materials and Methods

### 4.1. Sample Collection

**Figure 6:**
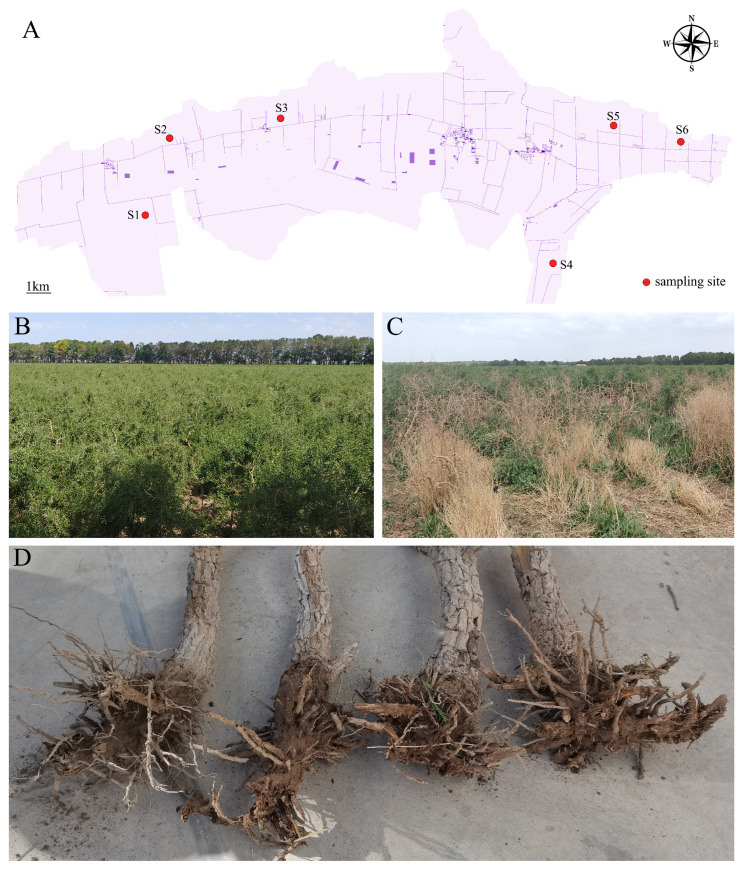
Map of the sampling site. (**A**) Geographic location of the six sampling sites; (**B**) the healthy *L. barbarum* in the field; (**C**) the root-rot *L. barbarum* in the field; (**D**) the decayed roots of *L. barbarum*.

Soil samples were collected in July 2023 from the Nomuhong Farm (36°26′21.67″ N, 96°25′55.42″ E) located in Dulan County, Qinghai Province (Figure 6A). The sampling site is at an approximate altitude of 2800 m above sea level. It is situated within the Qaidam Basin, a core production area for *L. barbarum* in China. The region is characterized by a hyper-arid climate with an average annual precipitation of approximately 15 mm and features typical sandy soil. Six healthy plants, showing no visible symptoms of disease (Figure 6B), and six plants exhibiting typical root rot symptoms (e.g., yellowing leaves, root decay; Figure 6C) were randomly selected for sampling. For each plant, bulk soil was first removed from the root system. The roots were then shaken to dislodge loosely attached soil, and the remaining tightly adhering soil, defined as the rhizosphere soil, was collected using a sterile brush [41]. Each plant was treated as an independent biological replicate. The collected soil samples were immediately placed on dry ice for transport and subsequently stored in a −80 °C ultra-low-temperature freezer until further processing.

### 4.2. DNA Extraction, PCR Amplification, and PacBio Sequencing

Total genomic DNA was extracted from the soil samples using the E.Z.N.A.^®^ soil DNA kit (Omega Bio-tek, Norcross, GA, USA) according to the manufacturer’s protocol. The integrity of the extracted DNA was assessed by electrophoresis on a 1% agarose gel, while its concentration and purity were quantified using a NanoDrop 2000 spectrophotometer (Thermo Scientific, Wilmington, DE, USA). High-quality genomic DNA served as the template for PCR amplification. The full-length bacterial 16S rRNA gene was amplified using the universal primers 27F (5′-AGRGTTYGATYMTGGCTCAG-3′) and 1492R (5′-RGYTACCTTGTTACGACTT-3′) [42]. The fungal internal transcribed spacer (ITS) region was amplified using the primers ITS1F (5′-CTTGGTCATTTAGAGGAAGTAA-3′) and ITS4R (5′-TCCTCCGCTTATTGATATGC-3′) [43]. Following purification of the PCR products, sequencing libraries were constructed using the SMRTbell Express Template Prep Kit 2.0. High-fidelity (HiFi) long-read sequencing was performed on a PacBio Sequel II platform by Shanghai Majorbio Bio-Pharm Technology Co., Ltd. (Shanghai, China).

### 4.3. Bioinformatic and Statistical Analysis

Raw sequencing data (subreads) generated by the PacBio platform were processed using the ccs (circular consensus sequencing) module of the SMRTLink software (v11.0) to generate high-quality HiFi reads. The reads were then demultiplexed based on barcode sequences, and primer sequences were trimmed. The QIIME 2 pipeline (v2022.2) was employed for downstream analysis. Specifically, the DADA2_CCS plugin was used to denoise the HiFi reads, correct sequencing errors, and generate a feature table of Amplicon Sequence Variants (ASVs) and their corresponding abundances, using default parameters adjusted for PacBio HiFi data. Taxonomic classification of the ASVs was performed using the classify-sklearn method in QIIME 2, which employs a pre-trained Naive Bayes classifier. The SILVA database (v138.1) was used for bacterial 16S rRNA gene classification, and the UNITE database (v8.3) was used for fungal ITS classification. The UNITE database was chosen as it is a highly curated reference set specifically for fungal ITS sequences, which is critical for accurate identification in mycological studies. Key ASVs were also queried against the NCBI nucleotide database to confirm classifications. To mitigate bias arising from variable sequencing depth, the ASV table was rarefied to the minimum sequence count across all samples prior to diversity analyses. Alpha diversity indices (Shannon, Simpson, Chao1) were calculated using Mothur (v1.30) to assess within-sample species richness and evenness. Beta diversity was assessed based on the Bray–Curtis dissimilarity metric. Community structure differences were visualized using Non-metric Multidimensional Scaling (NMDS), and the statistical significance of the separation between healthy and diseased groups was tested using Analysis of Similarities (ANOSIM).

### 4.4. Differential Abundance and Co-Occurrence Network Analysis

The Wilcoxon rank-sum test was used to identify microbial genera that were differentially abundant between the healthy (H) and diseased (D) sample groups. To explore inter-species interactions, co-occurrence networks were constructed based on the 100 most abundant ASVs in the fungal and bacterial datasets, respectively. Spearman’s rank correlation coefficients were calculated for all pairs of these ASVs using the psych package in R. The resulting correlation matrices were used to build the networks, which were visualized using Gephi (v0.9.2). Key topological parameters, including the number of nodes and edges, modularity, and average clustering coefficient, were calculated to evaluate the complexity and stability of the microbial communities.

### 4.5. Fungi Isolation, Identification, and Antagonism Assay

Fungi strains were isolated from soil samples using the serial dilution plating method on Potato Dextrose Agar (PDA). Briefly, 10 g of soil was suspended in 90 mL of sterile water and shaken vigorously. A dilution series (from 10^−2^ to 10^−5^) was prepared, and 100 µL aliquots from the 10^−3^, 10^−4^, and 10^−5^ dilutions were spread onto PDA plates. The plates were incubated at 25 °C for 3 days. Distinct colonies were selected and purified by inoculating on fresh PDA plates. Purified strains were preserved in 20% glycerol at −80 °C. *Fusarium* and *Trichoderma* strains were identified by their conidiophore and conidium morphological characteristics under a microscope [44,45]. We acknowledge that this preliminary identification to the genus level is a limitation. For definitive species-level identification required for developing targeted biocontrol strategies, future work should include molecular analysis of key phylogenetic markers. *Fusarium* and *Trichoderma* strains were then divided into different groups according to their colonies, conidiophores and conidia morphology. The antagonistic potential of *Trichoderma* groups against pathogenic *Fusarium* groups was evaluated using a four-point plate confrontation assay [46]. A mycelial plug of a *Fusarium* group was placed at the center of a PDA plate, and four mycelial plugs of a test *Trichoderma* group were placed symmetrically around it. Plates were incubated at 25 °C, and the radii of the *Fusarium* colonies were measured in both the confrontation plates and control plates (*Fusarium* only). The inhibition rate (I) was calculated using the formula:

I = R/(R − r) × 100%,(1)

where R is the colony radius of the pathogen in the control group and r is the colony radius of the pathogen in the confrontation group.

## 5. Conclusions

This study provides a strong ecological rationale for investigating a targeted biocontrol strategy against Goji berry root rot. The central finding is a competitive imbalance between pathogenic *Fusarium* and antagonistic *Trichoderma*, which correlates with the shift between a healthy and a diseased soil state. The most immediate practical application is to develop native *Trichoderma* strains, which demonstrated potent inhibition of *Fusarium* (averaging 75.3%), into localized bio-fungicides or soil amendments. Using indigenous strains offers a significant advantage in environmental adaptability and efficacy. It should be noted that the antagonism assay, while demonstrating strong inhibition, could be strengthened in future work by including a *Fusarium–Fusarium* co-culture control to account for any potential auto-inhibition. Nevertheless, the high rates of inhibition observed are strongly indicative of antagonistic activity by the Trichoderma isolates.

This research underscores that effective, long-term disease management should move beyond eliminating a single pathogen and focus on restoring a complex, stable, and disease-suppressive soil microbiome. Future research should prioritize (1) the species-level molecular identification of the most effective antagonistic *Trichoderma* and pathogenic *Fusarium* strains, (2) conducting field trials to validate the efficacy of these biocontrol agents under production conditions, and (3) integrating their application with other sustainable practices like organic matter amendment and crop rotation to rebuild soil health.

## Figures and Tables

**Table 1:** ITS Sequencing Data.

Amplicon	Sample Name	Raw Reads	Clean Reads	Base Number	Average Length	ASVs Number
Fungi	H (Mean ± SD)	27,754.7 ± 49	26,965.3 ± 46	16,554.8 ± 27	616.8 ± 14.2	97.7 ± 10.3
	D (Mean ± SD)	36,189.2 ± 12	34,625.8 ± 11	22,254.2 ± 70	637.7 ± 15.1	133.0 ± 68.3
Bacteria	H (Mean ± SD)	27,562.7 ± 46	27,479.5 ± 46	41,085.9 ± 68	1489.0 ± 2.2	359.0 ± 113.8
	D (Mean ± SD)	29,064.0 ± 71	28,993.2 ± 71	46,249.5 ± 11	1491.8 ± 6.0	459.5 ± 204.4

**Table 2:** Alpha Diversity Indices of Fungal and Bacterial Communities.

Amplicon	Estimators	*p*-Value	D-Mean	H-Mean
Fungi	Shannon	0.6584	2.9509	3.1076
	Simpson	0.4085	0.1372	0.1028
	Chao	0.2808	131.0429	96.1250
Bacteria	Shannon	0.5676	5.3806	5.4460
	Simpson	0.8796	0.0052	0.0051
	Chao	0.2848	342.6116	427.9333

**Table 3:** Topological features of ASV-level networks for healthy (H) and diseased (D) samples.

	Fungus (D)	Fungus (H)	Bacteria (D)	Bacteria (H)
Node	100	100	46	98
Edge	289	292	99	449
Positive Correlation Rate	81.16%	83.74%	51.52%	50.11
Negative Correlation Rate	18.84%	16.26%	48.48%	49.89
Average Degree	2.89	2.92	2.152	4.582
Graph Density	0.021	0.029	0.048	0.047
Modularity	0.413	0.818	0.554	0.514
Average Clustering Coefficient	0.347	0.379	0.236	0.242

## Data Availability

The original contributions presented in this study are included in the article. Further inquiries can be directed to the corresponding author.
